# Esketamine-induced dentate gyrus plasticity in treatment resistant depression: first-in-human evidence

**DOI:** 10.1192/j.eurpsy.2026.11630

**Published:** 2026-07-31

**Authors:** A. Le, Berre, D. Attali, I. Uszynski, P. Piche, C. Debacker, M. Lui, S. Charron, C. Paolillo, M. Moyal, F. Ramon, A. Henensal, J. Benzakoun, M. Danon, L. Mekaoui, P. Gorwood, C. Poupon, A. Cachia, C. Oppenheim, M. Plaze

**Affiliations:** 1 Université Paris Cité, Institute of Psychiatry and Neuroscience of Paris (IPNP), INSERM U1266, Ima-Brain Team; 2 GHU Paris Psychiatrie et Neurosciences, Hôpital Sainte-Anne, Service d’Imagerie diagnostique et thérapeutique; 3 GHU Paris Psychiatrie et Neurosciences, Hôpital Sainte-Anne, Unité de Neuropsychiatrie interventionnelle; 4 Physics for Medicine Paris, Inserm U1273, CNRS UMR 8063, ESPCI Paris, PSL University, Paris; 5 BAOBAB, UMR 9027, NeuroSpin, Université Paris-Saclay , CNRS, CEA, Gif-sur-Yvette; 6 Université Paris Cité, Laboratoire de Psychologie du Développement et de l’Education, UMR CNRS; 7 GHU Paris Psychiatrie et Neurosciences, Hôpital Sainte-Anne, Centre de référence des maladies rares à expression psychiatrique, pôle PEPIT; 8 Université Paris Cité, Institute of Psychiatry and Neuroscience of Paris (IPNP), INSERM U1266, “Vulnerability of Psychiatric and Addictive Disorders” Team; 9 GHU Paris Psychiatrie et Neurosciences, Hôpital Sainte-Anne, CMME, Paris; 10 BAOBAB, UMR 9027, NeuroSpin, Université Paris-Saclay , CNRS, CEA, Gif-sur-Yvette, France

## Abstract

**Introduction:**

Treatment-resistant depression (TRD) is marked by impaired hippocampal synaptic plasticity, potentially reversible with intranasal esketamine (ESK). Preclinical studies have demonstrated robust synaptic potentiation and dendritic spine growth in rodent hippocampus after ESK, but in-human evidence is lacking.

**Objectives:**

To detect early post-ESK microstructural changes in the dentate gyrus (DG) using advanced diffusion MRI in TRD patients, and to assess whether baseline MRI metrics predict clinical response.

**Methods:**

Twelve adults with TRD and 24 matched healthy controls were enrolled (ethics-approved; ClinicalTrials.gov NCT06661616). TRD patients were assessed at baseline (V1), 2 weeks after ESK initiation (V2), and and at treatment completion (V3). Depression severity was rated with MADRS. MRI (3DT1, multi-shell diffusion) was acquired at V1 and V2 in TRD, and at V1 in controls. DG volumes were computed with FreeSurfer; conventional diffusion metrics and Neurite Orientation Dispersion and Density Imaging (NODDI) metrics were extracted with Ginkgo. Linear mixed-effects models tested effects of group, time, age, and sex; Pearson correlations examined associations between MRI metrics and MADRS improvement. P-values were FDR-corrected.

**Results:**

Between V1 and V2, DG volumes remained stable while right DG fractional anisotropy (FA) decreased (χ² = 9.38, PFDR = 0.01) and left-DG orientation dispersion index (ODI) increased (χ² = 10.65, PFDR = 0.003) (Figure 1). No other hippocampal measures showed changes with time. Lower baseline FA in left DG correlated with greater symptom improvement at V2 (r=–0.57, p=0.05) (Figure 2), and FA decrease from V1 to V2 correlated with symptom improvement at V2 (r=0.74, p=0.01). No significant correlations were observed at V3.

**Image 1:**
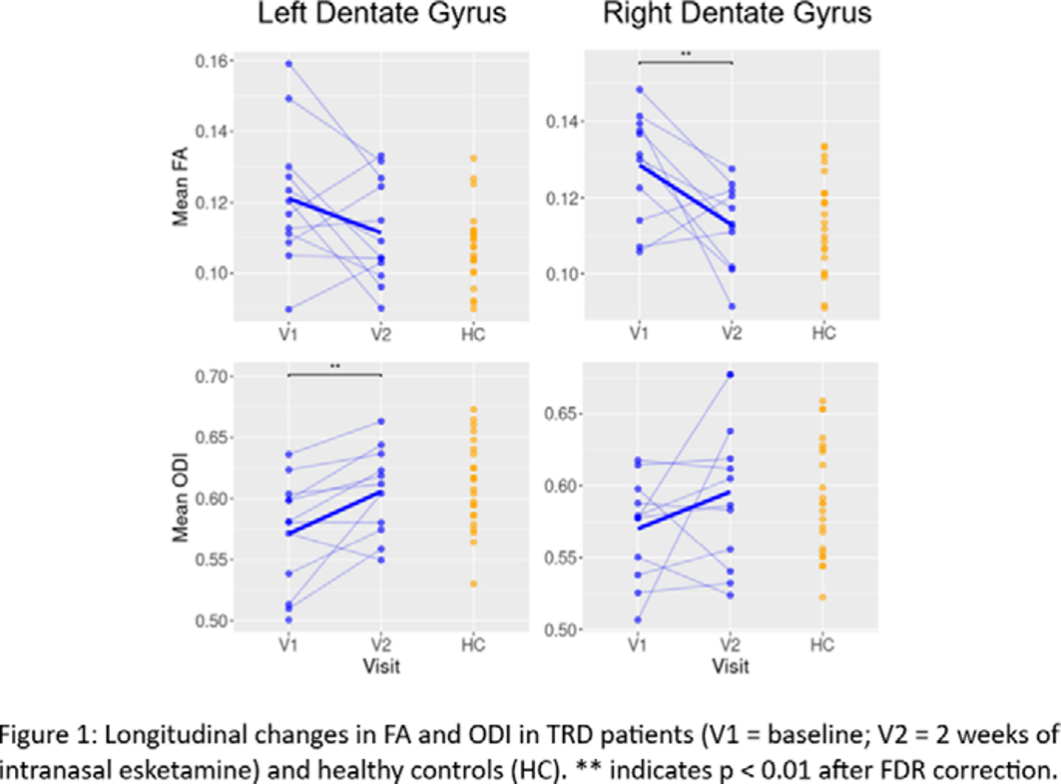
Image 1: Long description.

**Image 2:**
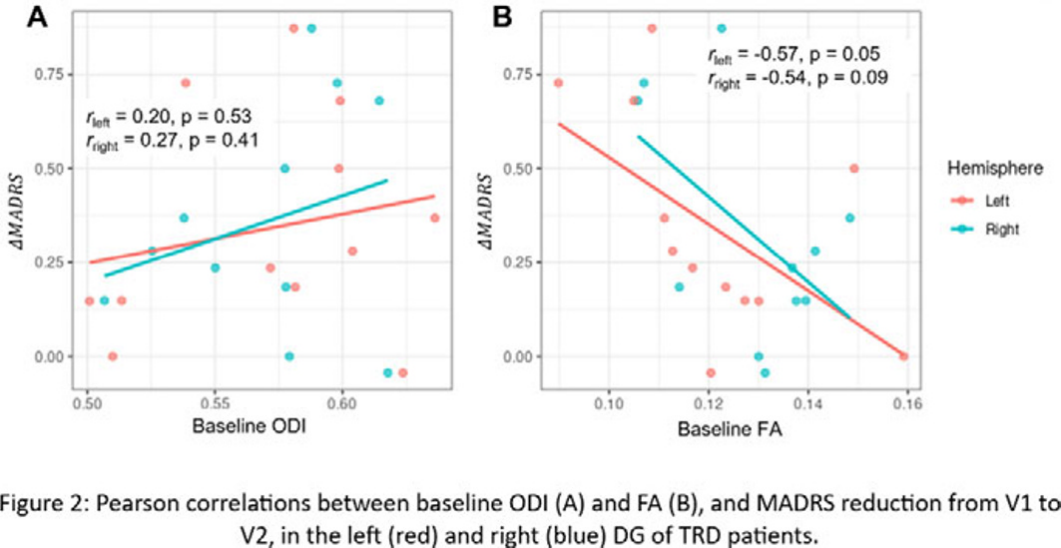
Image 2: Long description.

**Conclusions:**

ESK was associated with early microstructural remodeling in the human DG, reflected by decreased FA and increased ODI after two weeks of treatment, without detectable volumetric changes. This pattern - reduced FA and increased ODI - is consistent with increased dendritic complexity. Baseline DG diffusion metrics correlated with clinical improvement, suggesting their potential as in vivo proxies of synaptogenesis and ultimately as candidate biomarkers to guide TRD treatment. These first-in-human data support further validation in larger, longer-term cohorts.

**Disclosure of Interest:**

A. Le Berre Grant/Research from: received a research fellowship grant from the Servier Institute., D. Attali Consultant/speaker’s bureau/advisory activities: has been invited to scientific meetings, served as a speaker, and received compensation from Janssen. DA has been invited to scientific meetings by Neuraxpharm., I. Uszynski: None Declared, P. Piche: None Declared, C. Debacker: None Declared, M. Lui: None Declared, S. Charron: None Declared, C. Paolillo: None Declared, M. Moyal: None Declared, F. Ramon: None Declared, A. Henensal: None Declared, J. Benzakoun: None Declared, M. Danon Financial support from: received meals from Janssen-Cilag and Eisai SAS., L. Mekaoui Speakers Bureau of: served as a speaker and received compensation from Johnson & Johnson., P. Gorwood Consultant/speaker’s bureau/advisory activities: received during the last 5 years fees for presentations at congresses or participation in scientific boards from Biogen, Johnson & Johnson, Lundbeck, MindMed, MS Pharma, Newron, Otsuka, Richter and Viatris., C. Poupon: None Declared, A. Cachia: None Declared, C. Oppenheim Consultant/speaker’s bureau/advisory activities: served as speaker for Canon medical and Guerbet and received compensation fees for a scientific advisor board from Olea Medical., M. Plaze Consultant/speaker’s bureau/advisory activities: has been invited to scientific meetings, served as a speaker, and received compensation from Janssen.

## Long descriptions

### Image 1: Long description

The four panels are arranged in a two-by-two grid. The top row displays Mean F A (Fractional Anisotropy) and the bottom row displays Mean O D I (Orientation Dispersion Index). The left column represents the Left Dentate Gyrus and the right column represents the Right Dentate Gyrus.

* Top-Left Panel (Left Dentate Gyrus, Mean F A): The y-axis ranges from 0.08 to 0.16. Individual blue lines connect data points from V 1 to V 2, showing a general downward trend. A thick blue line indicates the mean decrease. To the right, an orange scatter plot shows H C (Healthy Control) values clustered between 0.09 and 0.13.

* Top-Right Panel (Right Dentate Gyrus, Mean F A): The y-axis ranges from 0.08 to 0.16. Blue lines show a significant decrease from V 1 to V 2, marked by a horizontal bracket with two asterisks. The mean thick blue line slopes downward. H C values are clustered between 0.09 and 0.14.

* Bottom-Left Panel (Left Dentate Gyrus, Mean O D I): The y-axis ranges from 0.50 to 0.70. Blue lines show a significant increase from V 1 to V 2, marked by a horizontal bracket with two asterisks. The thick blue mean line slopes upward. H C values are clustered between 0.53 and 0.67.

* Bottom-Right Panel (Right Dentate Gyrus, Mean O D I): The y-axis ranges from 0.50 to 0.70. Blue lines show a general upward trend from V 1 to V 2, with the thick blue mean line sloping upward. H C values are clustered between 0.52 and 0.66.

In all panels, V 1 and V 2 represent longitudinal visits for patients, while H C represents a static control group.

Navigate back to Image 1:.

### Image 2: Long description

A two-panel scatter plot with linear regression lines. A legend on the far right indicates that red dots and lines represent the Left hemisphere, while teal dots and lines represent the Right hemisphere. Both panels use delta M A D R S on the Y axis with a scale from 0.00 to 0.75.

Panel A: The X axis is Baseline O D I ranging from 0.50 to 0.65. The data shows a weak positive linear increase for both hemispheres. Statistics listed are r sub left equals 0.20, p equals 0.53 and r sub right equals 0.27, p equals 0.41.

Panel B: The X axis is Baseline F A ranging from 0.10 to 0.16. The data shows a moderate negative linear decrease for both hemispheres. Statistics listed are r sub left equals minus 0.57, p equals 0.05 and r sub right equals minus 0.54, p equals 0.09. The regression lines start at the top left and descend toward the bottom right, with the teal line for the right hemisphere appearing slightly steeper than the red line for the left hemisphere.

Navigate back to Image 2:.

